# Effects of inorganic and compost tea fertilizers application on the taxonomic and functional microbial diversity of the purslane rhizosphere

**DOI:** 10.3389/fpls.2023.1159823

**Published:** 2023-04-20

**Authors:** Angel Carrascosa, Jose Antonio Pascual, Álvaro López-García, María Romo-Vaquero, Ana De Santiago, Margarita Ros, Spyridon A. Petropoulos, Maria Del Mar Alguacil

**Affiliations:** ^1^ CSIC-Centro de Edafología y Biología Aplicada del Segura, Department of Soil and Water Conservation, Murcia, Spain; ^2^ Instituto Interuniversitario de investigación del Sistema Tierra en Andalucía, Universidad de Jaén, Jaén, Spain; ^3^ CSIC-Centro de Edafología y Biología Aplicada del Segura, Department of Food Science and Technology, Campus de Espinardo, Murcia, Spain; ^4^ Centro de Investigaciones Científicas y Tecnológicas de Extremadura (CICYTEX), Área de Protección Vegetal, Subárea de gestión y usos de suelos agrícolas y forestales, Instituto de Investigación Finca la Orden, Badajoz, Spain; ^5^ Department of Agriculture, Crop Production and Rural Environment, University of Thessaly, Volos, Greece

**Keywords:** *Portulaca oleracea*, crop, nitrogen application, Illumina sequencing, bacterial community, fungal community, functional composition

## Abstract

**Introduction:**

Soil fertility is a major determinant of plant-microbial interactions, thus, directly and indirectly affecting crop productivity and ecosystem functions. In this study, we analysed for the first time the effects of fertilizer addition on the cropping of purslane (*Portulaca oleracea*) with particular attention to the taxonomic and functional characteristics of their associated soil microbiota.

**Methods:**

We tested the effects of different doses of inorganic fertilization differing in the amount of N:P:K namely IT1 (300:100:100); IT2 (300:200:100); IT3 (300:200:200); and IT4 (600:100:100) (ppm N:P:K ratio) and organic fertilization (compost tea) which reached at the end of the assay the dose of 300 ppm N.

**Results and discussion:**

Purslane growth and soil quality parameters and their microbial community structure, abundance of fungal functional groups and prevailing bacterial metabolic functions were monitored. The application of compost tea and inorganic fertilizers significantly increased the purslane shoot biomass, and some soil chemical properties such as pH and soil enzymatic activities related to C, N and P biogeochemical cycles. The bacterial and fungal community compositions were significantly affected by the organic and chemical fertilizers input. The majority of inorganic fertilization treatments decreased the fungal and bacterial diversity as well as some predictive bacterial functional pathways.

**Conclusions:**

These findings suggest that the inorganic fertilization might lead to a change of microbial functioning. However, in order to get stronger evidence that supports the found pattern, longer time-frame experiments that ideally include sampling across different seasons are needed. Thus, further research is still needed to investigate the effects of fertilizations on purslane productivity under commercial field conditions.

## 1 Introduction

Mediterranean agro-ecosystems host a high diversity of wild edible plants (WEPs) that have been always an important food source and basic ingredients for the rural communities (Chatzopoulou et al., 2020; Corrêa et al., 2020). Currently, the increasing demand for healthy food and natural antioxidants from Central and Northern European countries, combined with the development of local cuisine, has rekindled the interest for the culinary use of WEPs in the so called “Mediterranean diet” (Sánchez-Mata et al., 2012; Ceccanti et al., 2018). Among them, purslane (*Portulaca oleracea* L.) is acknowledged as one of the most important medicinal and nutritional herbs because of its high content in omega-3 fatty acid (α-linolenic acid) and antioxidants (Petropoulos et al., 2015; Hosseinzadeh et al., 2021). Purslane is a palatable vegetable crop with mild flavour which can be consumed raw or cooked, typically in Turkey and other Mediterranean countries, and also in Asia (Kaymak, 2013).

To achieve the world agricultural needs, the increases in crop yields have been addressed through the application of chemical and organic fertilizers (Bebber and Richards, 2022; Dincă et al., 2022). Numerous studies have shown the negative effects of the long-term application of chemical fertilizers, especially nitrogen fertilizers, e.g. nitrate pollution of groundwater, eutrophication of surface waters, soil acidification or increasing greenhouse gas emissions (Singh and Craswell, 2021). Moreover, the excessive application of inorganic fertilizers has been recognized to be the most important factor contributing to the loss of soil health, mostly related to the loss of soil organic matter, the degradation of soil structure, and the decrease of soil biological activity (Zhao et al., 2016; Tian et al., 2017; Bebber and Richards, 2022; Dincă et al., 2022). Thus, it is urgent to find strategies to control and mitigate nitrogen loss from agricultural systems as well as to define the appropriate and optimized N application doses for each crop (Stark and Richards, 2008).

Conversely, organic fertilizers are known to improve soil health (Lewu et al., 2020), as well as the abundance, diversity and function of the soil microbial community (Jannoura et al., 2014; Insam et al., 2015; Bebber and Richards, 2022). These aspects make organic fertilizers a sustainable and environmentally friendly alternative to inorganic fertilization. Between organic fertilizers, compost teas (CTs) are oxygenated compost water extracts obtained through a suitable liquid-phase blowing process, that may improve the plant physiological status by increasing the crop yield and quality (Shaheen et al., 2013; Ros et al., 2020), while it could to be related to the reactivation and enhancement of the rhizosphere microbial communities that improve the plant nutrient uptake (Luo et al., 2022).

Soil microbial communities are considered to play a vital role in ecosystem functions, maintaining soil health, sustainability and productivity and regulating key processes, such as the carbon and nitrogen cycles (Mercado-Blanco et al., 2018). In fact, soil microorganisms have essential roles in organic N biosynthesis, N fixation, ammonification, nitrification, and denitrification (Kuypers et al., 2018; Hörstmann et al., 2021). Microbial communities are also determinants in promoting the plant growth and disease control and, consequently, the crop production (Saleem et al., 2019). It has been well documented that soil fertilization management influences the plant-microbial interactions and thus, the soil fertility and crop productivity (Sun et al., 2015; Pan et al., 2020; Ren et al., 2021; Dincă et al., 2022). Previous studies have found that fertilization changes the diversity, community structure and activity of soil microorganisms (Wei et al., 2017; Chen et al., 2019; Bebber and Richards, 2022; Dincă et al., 2022). In general, these studies have suggested that mineral fertilization, in particular N addition, decreases microbial diversity including plant-beneficial microbial taxa (Francioli et al., 2016; Cui et al., 2020). By contrast, the organic fertilization provides more stable conditions that favour the increase of microbial abundance and activity, probably due to an improvement of the soil structure and organic matter content (Zhan et al., 2018; Enebe and Babalola, 2020). Nevertheless, the beneficial effects of organic fertilization on the plant-soil system may vary depending on the fertilizer form, its nutrient content (NPK), soil structure, soil type, pH, crop type, etc. (Dincă et al., 2022).

In spite of numerous studies analysing the responses of the soil microbial communities to organic and chemical fertilizers for different crops (Dincă et al., 2022), the functional aspects of these impacts on bacterial and fungal communities have been scarcely investigated. Moreover, the effect of the addition of chemical and organic (such as compost tea) fertilization on the microbial community associated to purslane rhizosphere is completely unknown. In order to select a suitable management strategy for the establishment of a more stable and sustainable agroecosystems (Pan et al., 2020), it is of vital importance to understand the role that microbiota of purslane rhizosphere plays after the application of chemical and organic fertilizers.

We hypothesized that the addition of chemical and organic fertilizers (based on compost tea) on the short-term could alter the structure and function of the soil rhizosphere microbial community and promote the purslane growth and that this change could be dependent on the type and doses of fertilizer. In order to verify this hypothesis and to gain further insights into the relationship between fertilization and purslane crop, we assessed the effects of different doses of inorganic fertilization (NPK) compared to organic fertilization (compost tea) on the microbial community structure, the abundance of the fungal functional groups and the prevailing bacterial metabolic functions including functional genes associated with nitrogen cycling. Likewise, the changes in the soil quality properties and purslane growth parameters mediated by the different fertilization treatments were also evaluated.

## 2 Materials and methods

### 2.1 Design of the experiment

The topsoil (0–20 cm) used in the experiment came from the CEBAS-CSIC Experimental Farm (Santomera, Murcia, Spain 38°06’14.0”N 1°02’00.1”W), corresponding to a semi-arid Mediterranean climate with 19.2 °C as annual mean temperature, 300 mm annual rainfall and a high potential evapotranspiration of 1000 mm y^-1^. The soil, classified as Lithic xeric haploxeroll, is stony and shallow, with a clay-loam texture (clay fraction: 41% illite, 17% smectite, and 30% palygorskite). The chemical characteristics of the soil were: total N 05.7 g kg^−1^, available P 6.0 mg kg^−1^, available K 33.9 mg kg^−1^, organic matter 12.6 g kg^−1^, CaCO_3_ 3 g kg^−1^, pH 7.14 and EC 0.10 dS m^-1^.

The compost tea (organic fertilizer) was prepared weekly by mixing compost with distilled water in the ratio of 1:200 w/v after agitation for 2 h at ambient temperature. The raw materials to produce the compost were three at the following proportions: 45.6% vine pruning, 20.8% leek waste and 33.7% olive mill waste. The water extracts were filtered through a cheesecloth and stored at 4 °C until further use. The chemical properties of the compost tea were the following: total N 18.1 mg L^-1^, total C 69.06 mg L^-1^, C/N 3.8, P 5.8 mg L^-1^, K 99.8 mg L^-1^, Ca 23.6 mg L^-1^, Na 8.02 mg L^-1^, pH 8.33 and EC 0.45 dS m^-1^.

The growth substrate consisted of a mixture of sand, vermiculite and the above mentioned soil (1:1:1; w:w:w). Two kilograms of natural soil were placed in 2-litre pots (15.7-cm diameter, 12.5-cm height). In April 2021, seeds of purslane (*Portulaca oleracea* L.) provided by the Department of Agriculture Crop Production and Rural Environment (University of Thessaly, Greece) were directly sown in the pots in order to obtain one plant per pot after thinning. The experiment consisted of a mesocosm assay with four inorganic fertilization treatments which differed in the amount of N:P:K namely IT1: 300:100:100; IT2: 300:200:100; IT3: 300:200:200; IT4: 600:100:100 (ppm N:P:K ratio); one treatment of compost tea which reached at the end of the assay the dose of 300 ppm N; and one non-fertilizer control treatment. Each treatment was replicated in seven pots (n=7), making a total of 42 pots. The treatments were applied *via* nutrient solution to the pots, starting when plants developed 3-4 true leaves, e.g. May 2021. All the plants received 100 mL of nutrient solution per pot by manual irrigation once a week.

The experiment was maintained for three months (from seed sowing) under greenhouse conditions at CEBAS-CSIC Experimental Farm (Santomera, Murcia, Spain). Day/night temperature was 35 °C/25 °C. Plants were irrigated regularly with water to keep growth substrate at 60% of field capacity.

At experiment completion, plants were harvested and soil samples were collected. For each pot, the root system was placed into polyethylene bags and gently shaken to collect the rhizosphere soil. One portion of the rhizosphere soil was stored at -20 °C for DNA sequencing. The remaining soil was sieved through a 2-mm mesh for chemical and biochemical analyses. Fresh samples of shoots were also collected for further analyses.

### 2.2 Analyses of plant tissues

Fresh and dry (at 105 °C for 5 h) shoot biomass was measured. Dried shoots were ground with a mill before nutrient analysis. Shoot N was determined using a TruSpec CN Analyzer (LECO, St. Joseph, MI, USA). Shoot P and K contents were analysed by ICP/OES (Thermo Elemental Co. Iris Intrepid II XDL).

### 2.3 Chemical, biochemical, and biological analyses of rhizosphere soil

Soil pH and electrical conductivity were determined at 1:2.5 and 1:5 soil/water ratios, respectively. Available P was extracted with bicarbonate according to Olsen (1954) method and determined colorimetrically (viz., by using molybdate‐reactive P according to Murphy and Riley (1962)). Organic C (%) was quantified by dichromate oxidation method (Walkley and Black, 1934). Organic C values were transformed into organic matter by multiplication using an empirical factor (1.724). Total nitrogen (TN) and total carbon (TC) were determined using an elemental CHNS-O analyzer (EA- 1108, Carlo Erba, Barcelona, Spain); total phosphorous (TP) and total potassium (TK) using inductively coupled plasma-mass spectrometry (ICP-MS; ICAP 6500 DUO, Thermo Fisher Scientific, Hayward, California, USA). β-glucosidase activity (β-Glu) was assessed by using 25 mmol L^−1^ p-nitrophenyl-β-D-glucopyranoside as substrate according to Eivazi and Tabatabai (1988), and dehydrogenase activity (DHA) by measuring the amount of triphenyl formazan (TPF) released by incubating the soil with 2,3,5 triphenyltetrazolium chloride (Tabatabai, 1994). Urease activity was determined as described by Kandeler et al. (1999) using urea as substrate (Nannipieri et al., 1980). Alkaline phosphomonoesterase activities were determined colorimetrically as the p-nitrophenol released during the incubation (at adequate pH) of soil with the substrate p-nitrophenyl phosphate disodium (Naseby and Lynch, 1997).

### 2.4 DNA extraction and Illumina sequencing

Genomic DNA was extracted from 0.25 g of rhizosphere soil from each sample by using the DNeasy PowerSoil DNA Isolation kit (Qiagen), following the manufacturer protocol. DNA yield and quality were checked both by electrophoresis in 0.8% (*w*/*v*) agarose gels stained with GelRed and visualized under UV light, and by using a Qubit 3.0 fluorometer (Life Technologies, Grand Island, NY). DNA from each individual sample was sequenced using the Illumina MiSeq platform at the genomics service of the Institute of Parasitology and Biomedicine “López Neyra” (CSIC), Granada, Spain. Prokaryotic libraries were constructed by amplifying the hyper-variable V3–V4 regions of the 16S rRNA gene using the primer pair 341F (5′-CCTACGGGNBGCASCAG-3′) and 806R (5′-GACTACNVGGGTATCTAATCC-3′) according to Takahashi et al. (2014). These amplicons were tagged to be attached to PNA PCR clamps to reduce plastid and mitochondrial DNA amplification (Lundberg et al., 2013). Fungal libraries were constructed by amplifying the ITS2 region using the primer pair ITS4 (5′-TCCTCCGCTTATTGATATGC-3′) (White et al., 1990) and fITS7 (5′-GTGARTCATCGAATCTTTG-3′) (Ihrmark et al., 2012). Both runs were sequenced using a paired-end 2x300bp (PE 300) strategy.

### 2.5 Sequencing data processing

Raw sequence data was analyzed by following the DADA2 v. 1.16 analytical pipeline, adapted for ITS data in case of the fungal run (DADA2 ITS Pipeline Workflow v 1.8) (Callahan et al., 2016). Briefly, forward and reverse primers were removed using cutadapt (Martin, 2011). Reads were quality filtered and sequences dereplicated using standard settings in DADA2 pipeline. The rate error model was inferred and used to implement the sample inference algorithm. Forward and reverse reads were merged and chimeric sequences removed. To further curate for possible sequencing errors, we applied the LULU algorithm (Frøslev et al., 2017). In order to correct for mistagging, Amplicon Sequence Variants (ASVs) occurring at a frequency below 0.01% in each sample were removed. Taxonomic assignment was determined using the RDP algorithm implemented in DADA2 against the UNITE fungal database v. 8.2. (Abarenkov et al., 2022) for fungi and the 16S/18S SILVA release 132 for bacteria. ASVs not assigned to a known fungal phylum level were discarded as done for ASVs not assigned to the Bacteria kingdom for fungi and bacteria datasets, respectively. Finally, 1,227 ASVs (1,386,034 reads) and 3,204 ASVs (646,616 reads) were obtained for fungal and bacterial communities, respectively.

Fungal functional guilds were determined by matching fungal ASVs’ assigned genera and the genus-guild database Fungal Traits (Põlme et al., 2020). From this analysis, 838 ASVs (84.5% of reads) were assigned to a primary-lifestyle (sensu Põlme et al., 2020).

As a result, the ASVs were grouped into five fungal functional groups: Saprotrophs, Plant Pathogens, Parasites, Mycorrhizal fungi and Endophytes.

Bacterial metagenome prediction was performed on the basis of the ASV table using PICRUSt (Phylogenetic Investigation of Communities by Reconstruction of Unobserved States) (Langille et al., 2013). PICRUSt is used to estimate the bacterial genes present in the metagenomes of a microbial community under study, using 16S rRNA data, and to estimate the profile of the potential microbial functions associated with each of the groups. A normalization of each of ASV was carried out dividing each ASV by the known 16S copy number abundance. The functional profile of the genes was determined with the identification in Kyoto Encyclopedia of Genes and Genomes (KEGG) pathways (Kanehisa et al., 2012).

### 2.6 Statistical analyses

The normality and homogeneity of the variance of the environmental variables were checked using the Kolmogorov-Smirnov and the Levene’s tests, respectively. Data that were not normally distributed were transformed using a log transformation. One-way ANOVA was used to test the effect of the different treatments on measured variables and comparisons among means were made using the Tukey’s HSD (Honestly Significant Difference) test (*P* < 0.05).

To avoid bias in further analyses due to differences in sequencing effort between samples, the number of sequences per sample was rarefied to the lowest one when necessary. The rarefied ASVs table was used in further analyses.

A non-metric multidimensional scaling (NMDS) ordination based on the Bray-Curtis distance was performed to visually compare the taxonomic composition of the bacterial and fungal communities independently, and potential functional pathways of the bacterial communities, using the “metaMDS” function implemented in “vegan” package for R (Oksanen et al., 2019). To test the effect of the different treatments, on the rhizosphere microbial communities’ composition and structure, a permutational multivariate analysis (perMANOVA) using 999 permutations was conducted on the same Bray-Curtis matrix with the “adonis” function in vegan.

Canonical correspondence analyses (CCA) were carried out to quantify the relative importance of soil and plant properties in driving the changes in the composition of bacterial and fungal communities. Variance inflation factors (VIFs) were calculated to check the presence of collinearities among environmental variables using the function vif.cca in the “vegan” package and those with a VIF > 10 were sequentially removed. The remaining variables were then subjected to a forward selection procedure to select the subset of constraining variables that better explained the communities’ variation in the CCA final model. The significance of the CCA final models, for bacterial or fungal communities, was tested by Monte-Carlo permutational test (999 permutations). Heat maps correlations were constructed using R and vegan package.

The influence of the treatments on bacteria functional profiles were evaluated individually for each function by using the Kruskal-Wallis test. The Benjamini–Hochberg false discovery rate (FDR) (Benjaminit and Hochberg, 1995) correction method was applied to the results and all features with a p value ≥0.05 were removed.

### 2.7 Data availability

The sequences retrieved in this study were submitted to the NCBI Sequence Read Archive repository (www.ncbi.nlm.nih.gov/sra) and they are accessible through the BioProject PRJNA934288.

## 3 Results

### 3.1 Effects of the fertilization treatments on plant growth parameters

**Figure 1:**
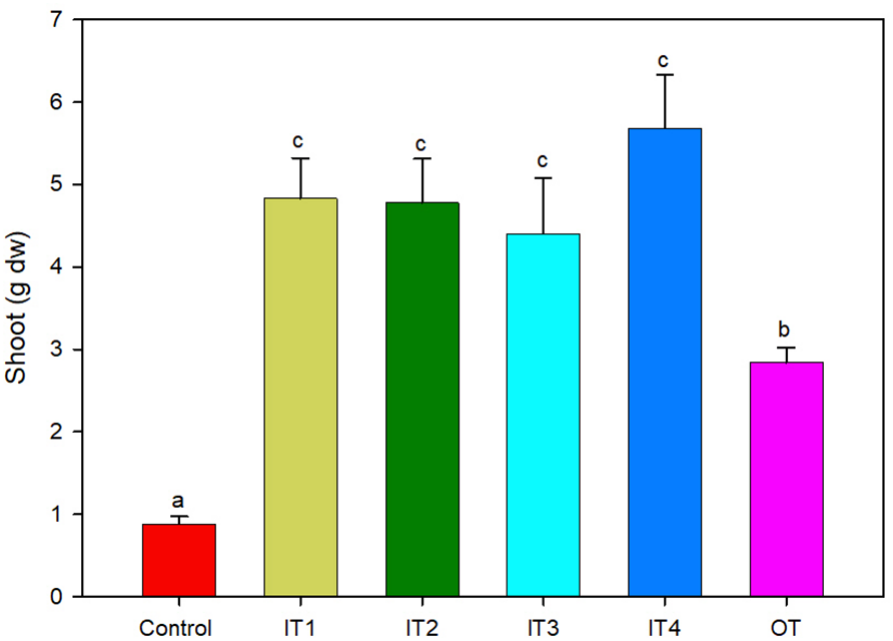
Shoot dry biomass of purslane plants grown under five different fertilization treatments (n = 4). For each treatment, bars followed by the same letter are not significantly different according to Tukey’s HSD-test (p < 0.05). Bars represent standard error.

The application of fertilization (inorganic and organic) increased significantly the plant biomass (fresh and dry) (**Figure 1** and **Table S1**). In particular, inorganic fertilizer treatments resulted in significantly higher fresh and dry shoot biomass, compared to the tea compost and the control treatment, while significant differences were also recorded between the organic fertilizer and the control.

The treatment with the highest N doses (IT4) showed the highest plant growth (84% respect to the control plants), as well as the highest N content. By contrast, the P leaf content was significantly decreased for all the fertilization treatments used in this study compared to the control, showing the lowest content in the IT4 treatment (higher N doses) and the highest content in the control plants.

### 3.2 Effects of the fertilization treatments on rhizosphere soil properties

The TC, TN, OC, OM and EC of rhizosphere soil were not significantly affected by the fertilization treatments (**Table 1**). The pH increased significantly by all fertilization treatments (inorganic and organic) compared to the control treatment. Only the IT2 treatment significantly increased the total P content in the soil compared to the rest of the treatments (except for IT1 treatment where no significant differences were detected).

**Table 1:** Chemical, physico-chemical and biological properties of the rhizosphere of purslane plants grown under different fertilization treatments.

Treatments	Control	IT1	IT2	IT3	IT4	OT	ANOVA(F value (P value))
pH	8.44 ± 0.12 a	8.75 ± 0.05 b	8.85 ± 0.15 b	8.73 ± 0.09 b	8.77 ± 0.06 b	8.78 ± 0.08 b	**8.64 (<0.001)**
EC (µS cm^-1^)	93.48 ± 7.28	89.88 ± 11.68	95.33 ± 9.48	96.53 ± 15.19	72.93 ± 3.37	101.38 ± 13.76	**1.13 (ns)**
TN (g Kg^-1^)	0.46 ± 0.02	0.58 ± 0.21	0.66 ± 0.26	0.52 ± 0.15	0.70 ± 0.18	0.67 ± 0.14	**1.24 (ns)**
TC (g Kg^-1^)	16.36 ± 5.14	17.38 ± 5.79	17.32 ± 3.23	17.52 ± 4.02	17.08 ± 1.18	20.45 ± 3.35	**0.47 (ns)**
TP (g Kg^-1^)	0.36 ± 0.08 ab	0.46 ± 0.06 bc	0.55 ± 0.04 c	0.34 ± 0.05 ab	0.43 ± 0.1 b	0.28 ± 0.04 a	**6.22 (<0.01)**
TK (g Kg^-1^)	3.53 ± 0.65 ab	3.82 ± 0.25 b	3.72 ± 0.44 b	2.82 ± 0.57 a	3.63 ± 0.68 b	2.81 ± 0.42 a	**2.90 (<0.05)**
OC (g Kg^-1^)	3.78 ± 0.93	4.10 ± 1.51	5.20 ± 0.13	4.05 ± 1.64	3.67 ± 1.10	3.97 ± 0.47	**0.76 (ns)**
OM (g Kg^-1^)	6.51 ± 1.60	7.06 ± 2.60	8.96 ± 0.23	6.97 ± 2.82	6.33 ± 1.90	6.84 ± 0.81	**0.76 (ns)**
AP (g Kg^-1^)	0.19 ± 0.01 ab	0.22 ± 0.05 b	0.21 ± 0.03 b	0.23 ± 0.03 b	0.23 ± 0.03 b	0.16 ± 0.01 a	**2.93 (<0.05)**
GLC (µmol PNF g-1 h-1)	0.38 ± 0.05 a	0.49 ± 0.04 bc	0.58 ± 0.12 c	0.48 ± 0.07 bc	0.56 ± 0.01 c	0.43 ± 0.07 ab	**4.77 (<0.01)**
PHO (µmol PNF g-1 h-1)	1.00 ± 0.10 a	1.40 ± 0.09 b	1.28 ± 0.08 b	1.27 ± 0.07 b	1.62 ± 0.24 c	1.26 ± 0.14 b	**8.73 (<0.001)**
URE (µmol N-NH4+ g-1 h-1)	3.24 ± 0.56 a	5.21 ± 0.2 c	5.99 ± 0.87 cd	4.28 ± 0.21 b	6.36 ± 0.62 d	4.27 ± 0.70 b	**15.21 (<0.001)**
DHA (µg TPF g-1 h-1)	4.26 ± 0.53 a	6.46 ± 0.53 b	6.40 ± 0.36 b	6.10 ± 0.38 b	7.05 ± 1.01 b	4.48 ± 1.00 a	**10.35 (<0.001)**

Means ± standard deviation, n=4. Significance of effects of treatments on the measured variables is also shown (F-values (P-values)). Values in columns sharing the same letter do not differ significantly (P<0.05) as determined by the Tukey HSD test. EC (electrical conductivity), TN (soil total nitrogen), TC (soil total carbon), TP (soil total phosphorous), TK (soil total potassium), OC (soil organic carbon), OM (soil organic matter), AP (available phosphorous), GLC (β – glucosidase), PHO (alkaline phosphomonoesterase), URE (urease), DHA (Dehydrogenase). ANOVA, analysis of variance, ns: non-signifcant.

The AP was significantly higher in inorganic fertilizers (IT1, IT2, IT3, IT4) compared to OT. On the other hand, K content was higher in IT1, IT2 and IT4 than the IT3 and OT treatments, while no significant differences were recorded over the control.

In general, the fertilization treatments significantly affected the soil enzyme activities (**Table 1**). All inorganic fertilization treatments (IT1, IT2, IT3 and IT4) significantly increased the four enzymatic activities measured, while the alkaline phosphomonoesterase and urease activities increased significantly by all fertilization treatments, including inorganic and organic ones.

### 3.3 Effects of the fertilization treatments on the rhizosphere soil bacterial community

Sequencing of 16S amplicon of rhizosphere samples yielded a total of 2,110,216 raw sequences. After applying all the quality filters and cleaning criteria, we obtained 561,086 high-quality bacterial sequences across all the samples (on average, about 24,395 reads per sample), represented by 2,725 ASVs.

The rarefaction curves showed a full coverage of the bacterial diversity for every sample, indicating that the sequencing effort was similar and making the diversity across samples comparable (**Figure S1A**).

The bacterial richness (S) under the OT treatment was significantly higher than the control and inorganic treatments, except IT3 (**Table S2**). In the case of Shannon diversity index (H), the inorganic treatment with higher N doses (IT4) resulted in a significant decrease in relation to the control and the rest of the treatments.

**Figure 2:**
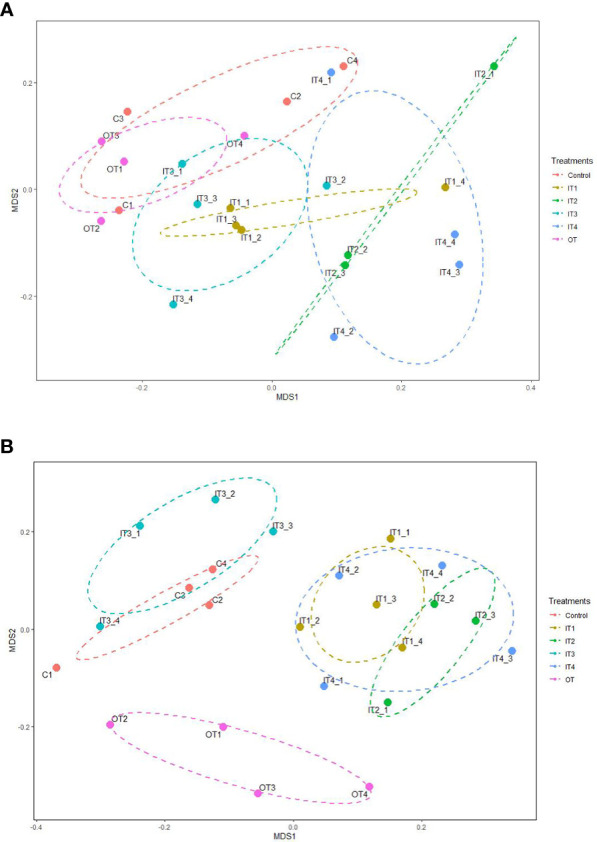
**(A)** Non-metric multidimensional scaling (NMDS) analysis on a Bray-Curtis dissimilarity matrix based on the ASVs dataset retrieved from the bacterial communities of the rhizospheric soil of purslane under different fertilization treatments. Ellipsoids represent 95% confidence for each treatment mean. **(B)** Non-metric multidimensional scaling (NMDS) analysis on a Bray-Curtis dissimilarity matrix based on the ASVs dataset retrieved from the fungal communities of the rhizospheric soil of purslane under different fertilization treatments. Ellipsoids represent 95% confidence for each treatment mean.

The perMANOVA analysis showed that fertilization treatments significantly affected the bacterial community composition of rhizosphere soil of purslane (*F*= 1.52, p= 0.001). The NMDS ordination plots showed that the bacterial communities belonging to the inorganic fertilization treatments were significantly different from the control and the organic treatment (Stress=0.13, *R* ^2 =^ 0.984) (**Figure 2A**).

A total of 25 bacterial phyla were found across all the rhizosphere soil samples from purslane. The most abundant phyla were *Proteobacteria* (23%), *Actinobacteria* (22%) and *Chloroflexi* (21%), followed by *Cyanobacteria* (13%), *Patescibacteria*, *Acidobacteria* and *Planctomycetes* (all three around 5%). The relative abundance of the remaining phyla was below 1.5% (**Figure S2A**). The treatment with the highest doses of N (IT4) decreased significantly the *Chloroflexi* and *Acidobacteria* phylum. On the other hand, the *Gemmatimonadetes* and *Entotheonellaeota* phylum were also significantly decreased for the inorganic fertilization treatments (**Figure S2A**).

### 3.4 Effects of the fertilization treatments on the rhizosphere soil fungal community

The ITS2 amplicon sequencing generated 2,595,367 raw reads. After applying the subsequent filtering and cleaning processes we obtained 905 fungal ASVs (572.447 sequences). The rarefaction curves calculated for the rarefied dataset (24.889 sequences per sample) indicated that the sequencing effort covered the whole ASV richness per sample (**Figure S1B**).

The richness (S) and Shannon diversity index (H) were significantly affected by the fertilization treatments (**Table S3**). Except for the organic (OT) and inorganic fertilization IT3 treatments, the rest of inorganic treatments significantly decreased the richness and diversity index of the fungal community.

Fertilization treatments had a significant effect on fungal community composition of rhizospheric soil of purslane (*F*= 2.01, p= 0.001), as was easily visualized in the NMDS ordination plot (**Figure 2B**). In this ordination analysis, three groups were clearly observed, the control and IT3 fungal communities, the other inorganic fertilizers (IT1, IT2, IT4) and the OT fungal communities (Stress=0.15, *R* ^2 =^ 0.975).

The fungal community was composed of 14 fungal phyla. The most abundant phylum was *Ascomycota* accounting for 65% of relative abundance, followed by *Mortierellomycota* (17%), *Basidiomycota* (8.7%), *Chytridiomycota* and *Olpidiomycota* (both phyla around 4%). The remaining phyla accounted for less than 1% of the fungal sequences (**Figure S2B**). Only the *Rozellomycota* phylum was significantly negatively affected by the inorganic fertilization treatments, except for the IT3 treatment where no significant effects were recorded (**Figure S2B**).

### 3.5 Effects of the fertilization treatments on predicted functions of rhizosphere soil bacterial and fungal communities

**Figure 3:**
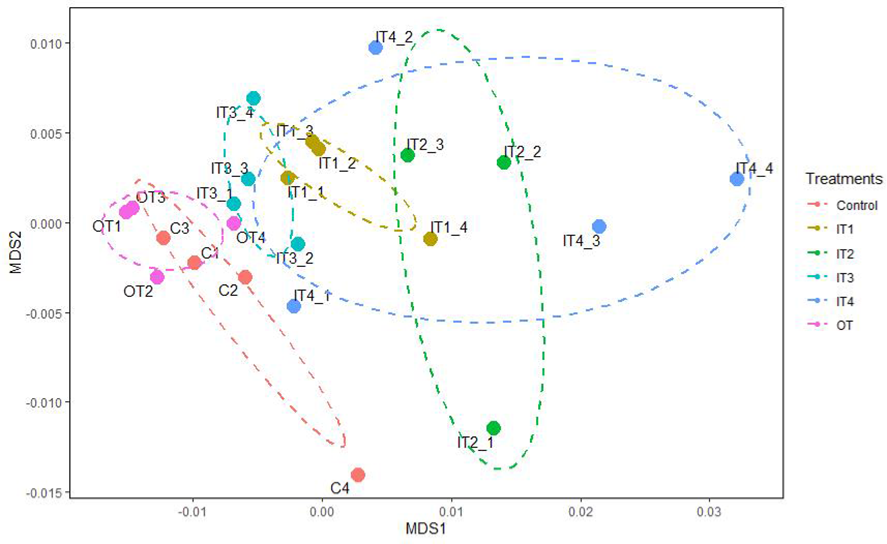
Non-metric multidimensional scaling (NMDS) analysis on a Bray-Curtis dissimilarity matrix based on the predicted functional pathway profiles associated with the bacterial communities of the rhizosphere of purslane under different fertilization treatments. Ellipsoids represent 95% confidence for each treatment mean.

Functional analysis using PICRUSt revealed a total of 35 significant pathways for the bacterial communities of the purslane rhizosphere under the different fertilization treatments (**Table S4**). In general, the chemical fertilization, mainly the IT2 and IT4 treatments decreased significantly the pathways related to cellular processes, membrane transport, genetic information processing and metabolism, such as: amino acid, carbohydrate, lipid and cofactors and vitamins. By contrast, the pathways related with the metabolism of terpenoids and polyketides and the biosynthesis and biodegradation of secondary metabolites were significantly increased by the inorganic fertilization (IT1, IT2 and IT4). The organic fertilization did not affect the predictive functions. These results can be observed also in the ordination plot of the NMDS analysis, where it is shown that this predictive functional profiling of the bacterial communities belonging to the inorganic fertilization was separated from both the organic fertilization and the control treatment (**Figure 3**). The influence of the treatments on the functional capabilities of the bacterial community was also corroborated by the perMANOVA analysis (*F*= 3.90, p= 0.001).

With respect to the N-cycling pathway genes (**Table 2**), we found that in the nitrification process, the ammonia oxidation-predicted genes (*amoA*, *amoB* and *amoC*) were significantly increased in the chemical treatments IT3 and IT4. The relative abundance of nitrite reductase predicted gen (*nirK*) belonging to denitrification process also was increased by the inorganic fertilization, being significantly affected by the IT2 and IT4 treatments. The rest of predicted N-cycling genes were not affected by the inorganic and organic fertilization.

**Table 2:** Predicted N metabolism pathway genes count detected under the five different fertilization treatments.

Treatment	N-fixation				Denitrification							Nitrification			
	*anfG*	*nifH*	*nifD*	*nifK*	*narG*	*narH*	*narI*	*nirK*	*norB*	*norC*	*nosZ*	*amoA*	*amoB*	*amoC*	*hao*
Control	0.26 ± 0.07	0.15 ± 0.02	0.15 ± 0.02	0.15 ± 0.02	0.95 ± 0.11	0.11 ± 0.01	0.51 ± 0.08	0.66 ± 0.07a	0.96 ± 0.03bc	0.20 ± 0.03	0.96 ± 0.16	0.12 ± 0.05a	0.12 ± 0.05a	0.22 ± 0.09a	0.45 ± 0.07
IT1	0.24 ± 0.05	0.13 ± 0.02	0.13 ± 0.01	0.13 ± 0.01	0.98 ± 0.07	0.11 ± 0.01	0.55 ± 0.08	0.71 ± 0.03ab	0.92 ± 0.09abc	0.18 ± 0.02	0.93 ± 0.05	0.27 ± 0.07ab	0.27 ± 0.07ab	0.49 ± 0.14ab	0.44 ± 0.03
IT2	0.19 ± 0.01	0.18 ± 0.08	0.16 ± 0.05	0.16 ± 0.05	0.85 ± 0.21	0.10 ± 0.02	0.49 ± 0.01	0.83 ± 0.10c	0.84 ± 0.06a	0.21 ± 0.04	1.13 ± 0.36	0.22 ± 0.03ab	0.22 ± 0.03ab	0.39 ± 0.05ab	0.37 ± 0.10
IT3	0.23 ± 0.04	0.13 ± 0.02	0.13 ± 0.02	0.13 ± 0.02	0.92 ± 0.07	0.11 ± 0.01	0.50 ± 0.05	0.74 ± 0.06abc	0.95 ± 0.04abc	0.21 ± 0.06	1.01 ± 0.06	0.35 ± 0.09b	0.35 ± 0.09b	0.63 ± 0.16b	0.44 ± 0.02
IT4	0.21 ± 0.03	0.17 ± 0.04	0.16 ± 0.03	0.16 ± 0.03	0.91 ± 0.16	0.10 ± 0.02	0.49 ± 0.09	0.81 ± 0.07bc	0.87 ± 0.09ab	0.22 ± 0.04	0.93 ± 0.22	0.38 ± 0.27b	0.38 ± 0.27b	0.68 ± 0.47b	0.42 ± 0.07
OT	0.30 ± 0.09	0.12 ± 0.01	0.13 ± 0.01	0.13 ± 0.01	0.93 ± 0.05	0.11 ± 0.01	0.51 ± 0.06	0.68 ± 0.07a	0.99 ± 0.05c	0.21 ± 0.05	0.94 ± 0.12	0.11 ± 0.03a	0.11 ± 0.03a	0.20 ± 0.05a	0.43 ± 0.04
**ANOVAF value(P value)**	**1.76(ns)**	**1.45(ns)**	**1.51(ns)**	**1.52(ns)**	**0.49(ns)**	**0.93(ns)**	**0.26(ns)**	**3.60(0.021)**	**2.64(0.05)**	**0.47(ns)**	**0.65(ns)**	**3.33(0.028)**	**3.33(0.028)**	**3.38(0.026)**	**0.83(ns)**

Data represent Mean and standard deviation (SD) for each treatment (n=4). Values are expressed with an e-value of 1x10^-1^ for nifH, nifD, nifK, narH genes, an e-value of 1x10^-2^ for norB, narI, nirK, narG, norC, nosZ, hao genes and an e-value of 1x10^-3^ for anfG, amoA, amoB, amoC genes. Values in columns sharing the same letter do not differ significantly (P<0.05) as determined by the Tukey HSD test. ANOVA, analysis of variance; ns: non-signifcant.

The fungal functional guilds analysis assigned 825 ASVs (67.23% of the total ASVs) to the main functional guilds: saprotrophs, parasites, plant pathogens, endophytes and mycorrhizal fungi (**Figure S3A**).

Only the relative abundances of mycorrhizal fungi and endophytes were significantly influenced by the fertilization treatments (*F*=3.44, *P*=0.027; *F*=9.433, *P*<0.001, for mycorrhizal fungi and endophytes, respectively). The relative abundance of endophytes decreased significantly with all fertilization treatments (organic and inorganic) with respect to the control treatment. In the case of mycorrhizal fungi, all the inorganic fertilization treatments (IT1, IT2, IT3 and IT4) decreased significantly (on average, about 76%) the relative abundance of sequences with respect to the control treatment (**Figure S3B**).

### 3.6 Relationship between the soil properties and plant parameters and the composition of the rhizosphere soil microbial communities

The forward selection procedure in CCA carried out for the rhizosphere bacterial community dataset revealed three variables (dehydrogenase activity, total K and foliar K) in the final CCA model, as shown in **Figure S4A**. This CCA final model explained 20.4% of the total inertia from which the first two axes explained a 14.8% of the total inertia.

In the case of the rhizosphere soil fungal communities, only urease activity was selected in the CCA procedure. This CCA final model explained 8% of the total inertia (**Figure S4B**).

**Figure 4:**
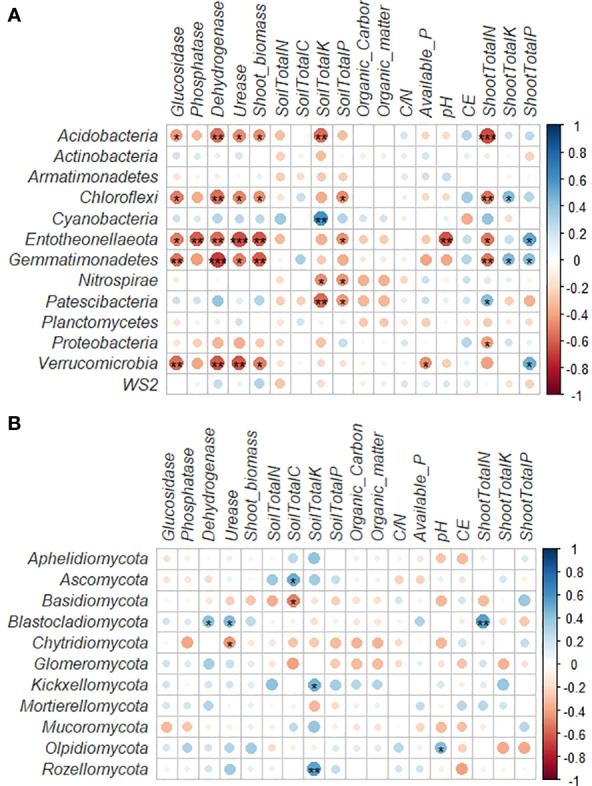
**(A)** Correlations between the dominant bacterial phyla and soil properties and plant parameters. The R value is shown in different colours in the figure. If the *P*-value is < 0.05, it is marked with an asterisk. *0.01 < P ≤ 0.05; **0.001 < P ≤ 0.01; ***P ≤ 0.001. **(B)** Correlations between the dominant fungal phyla and soil properties and plant parameters. The R value is shown in different colours in the figure. If the *P*-value is < 0.05, it is marked with an asterisk. *0.01 < P ≤ 0.05; **0.001 < P ≤ 0.01; ***P ≤ 0.001.

A correlation heat map generated to examine the relationships between the microbial phyla and soil and plant parameters revealed more notable variations among the bacterial phyla than fungal phyla (**Figures 4A, B**). Acidobacteria, Chloroflexi, Entotheonellaeota and Gemmatimonadetes showed significant negative correlations with the shoot nitrogen content (**Figure 4A**). Acidobacteria, Chloroflexi, Entotheonellaeota, Gemmatimonadetes and Verrucomicrobia also revealed significant negative correlations with plant biomass and soil enzymatic activities. Cyanobacteria only showed significant positive relation with the soil potassium content. Chloroflexi, Entotheonellaeota, Nitrospirae and Patescibacteria were negatively related with the soil phosphorous content. The soil pH only showed significant negative relation with Entotheonellaeota phylum. A positive significant relation with the plant phosphorous content was also found for Entotheonellaeota Gemmatimonadetes and Verrucomicrobia. Finally, Chloroflexi and Gemmatimonadetes showed positive significant relation with plant potassium content.

In the case of the fungal phyla, our analysis revealed that Blastocladiomycota showed significant positive correlations with the nitrogen content in plant and the urease and dehydrogenase enzymatic activities (**Figure 4B**). The soil carbon content was positively related with Ascomycota and negatively with Basidiomycota, while Kickxellomycota and Rozellomycota revealed significant positive relation with the soil potassium content.

## 4 Discussion

The results of this experiment showed that soils receiving different N doses of inorganic fertilization or compost tea as organic fertilization increased significantly the plant growth of purslane, particularly the inorganic fertilization treatments which resulted in the highest plant growth. This could be a result of the more efficient assimilation of nutrients from crops in the case of inorganic fertilization (Wang et al., 2017). Several studies have found that soils with N input exhibit greater microbial activities than soils receiving low-N fertilisation or no fertilization, and thus a higher production of extracellular enzymes involved in the biogeochemical cycles related to C, N and P transformations, (Sinsabaugh et al., 2014; Francioli et al., 2016; Murugan et al., 2019). In accordance, the rise of the rhizosphere microbial activity (urease, phosphatase, glucosidase and dehydrogenase) in response to fertilization with N was evidenced in our study under all the inorganic fertilization treatments (IT1, IT2, IT3 and IT4), while it improved slightly under the organic fertilization treatment (OT). Francioli et al. (2016) also found higher *N*-acetylglucosaminidase and phosphatase activities in mineral fertilized soils (NPK) compared to the organic fertilized ones indicating that mineral fertilization affects to a greater extent the enzymes involved in N and P cycling in soil. A possible explication for the increased enzymatic activities might be attributed to the higher biomass of the fertilized purslane plants that could induce the higher root exudation. In fact, in our study there was a strong positive significant correlation between plant biomass and glucosidase (r=0.62**), phosphatase (r=0.55**), dehydrogenase (r=0.70**) and urease (r=0.70**) activities. It has been suggested that increases in the aboveground (Geisseler and Scow, 2014; de Vries and Bardgett, 2016; Murugan et al., 2019) and belowground biomass (Pérès et al., 2013; Kumar et al., 2016) may promote changes in rhizodeposition patterns, which, in turn, can alter soil C storage that further influences belowground microbial processes (Murugan et al., 2019; Bebber and Richards, 2022).

On the other hand, these shifts in the microbial enzymatic activities related to nutrient cycles also could be explicated by alterations in the soil microbial community composition (Nannipieri et al., 2003). In our study, the organic treatment based on tea compost was the only one in which bacteria richness increased significantly; on the other hand, the bacterial diversity measured by Shannon diversity index was significantly decreased only for the plants that received the highest doses of N (e.g. IT4). Regarding the fungal diversity and richness, it was decreased by all the chemical fertilization treatments, except for the treatment IT3. In accordance, Wang et al. (2018b) suggested that the addition of other nutrients (P, K) along with N generally could mitigate the negative effects of N addition on microbial diversity to some degree. In general, the mineral fertilization reduced the diversity of soil microbial communities, being the fungal community more affected than bacterial community to N fertilizer inputs. Our findings are supported by previous studies (Wang et al., 2018a; Wang et al., 2018b; Ye et al., 2020; Dincă et al., 2022) and although there is currently no consensus on what drives this decrease in diversity, it could be related to changes in competition and/or adaptation processes, with fast-growing taxa that are able to take advantage of increased nutrients availability under fertilized soils outcompeting slower growing taxa better adapted to low nutrient conditions (Fierer et al., 2012a; Leff et al., 2015).

The NMDS ordination and PERMANOVA analyses revealed a significant effect of mineral and organic fertiliser applications on the soil microbial community structure.

Fertilizers addition altered fungal communities more strongly than bacterial ones a finding which is in agreement with previous studies indicating that fungi react more sensitively to nutrients addition than other microbial groups (Freedman et al., 2015; Widdig et al., 2020). We found that the fungal communities of the rhizosphere soil of purslane were completely different between organic and inorganic treatments. In the case of the bacterial communities, the organic fertilizer and control treatment shared the same community; being significantly different from that of the chemical fertilization treatments (IT1, IT2 and IT4). Therefore, alterations of the bacterial community distributions seem to be more closely related to chemical fertilization than organic fertilizer. Similarly to our results, several researchers also found differences in the rhizosphere fungal and bacterial communities composition with N fertilizer addition from different origin (inorganic and organic), both at long (Francioli et al., 2016; Wang et al., 2017; Pan et al., 2020; Widdig et al., 2020) and short-term (Paungfoo-Lonhienne et al., 2015; Enebe and Babalola, 2020; Craig et al., 2021). It has been suggested that variations in the soil microbial community composition are linked to the soil properties in a bidirectional way (van der Heijden et al., 2008). In this respect, we observed that the bacterial community composition was linked to foliar and soil potassium and dehydrogenase activity, while urease activity accounted for the shifts in the fungal community composition observed among the fertilization treatments. This is in agreement with other studies that have highlighted the relevance of soil properties to driving the rhizosphere microbial community in fertilized soils (Pan et al., 2020; Ren et al., 2021). The shifts in microbial community assembly induced by N fertilizers inputs from inorganic and organic origin may also be reflected in functional differences of the dominant taxa (phylum). Our results confirmed a decrease in predicted oligotrophic (K-strategists) groups (Chloroflexi, Acidobacteria and Gemmatimonadetes, Entotheonellaeota) with the addition of N (Zeng et al., 2016; Ling et al., 2017), mainly for the treatment with the highest N doses. This result was in line with the shifts in bacterial community composition following N manipulation that has been previously explained by the oligotrophic hypothesis (Fierer et al., 2012a), according to which oligotrophic bacteria have slow growth rates and their abundance could decrease in nutrient-rich conditions. In fact, we found a negative correlation between these bacterial phyla and soil nutrient content for the enzymatic activities related to nutrient cycles. Our findings were consistent with several studies carried out at long- (Yao et al., 2014; Eo and Park, 2016; Wang et al., 2018b; Ren et al., 2020) and short-term (Herzog et al., 2015) where the authors found a decrease of Acidobacteria with increased N fertilization. On the other hand, it has been suggested that soil pH has even a greater impact than N fertilization on soil microorganisms community structure and abundance (Zhao et al., 2014; Ling et al., 2017; Widdig et al., 2020; Chen et al., 2021), being Acidobacteria one of the phyla more influenced/correlated with this soil parameter (Fierer et al., 2012b; Liu et al., 2015; Francioli et al., 2016). By contrast, we did not find a correlation between the abundance of this phylum and soil pH. In our study, only the phylum Entotheonellaeota was negatively correlated with soil pH, suggesting that pH range in our fertilized soils does not provide insight into the relationship between soil pH and soil bacterial communities. The pH significantly increased under all treatments with application of inorganic and organic fertilizers. In general, pH tends to decrease with N addition and the majority of authors studying the effect of N mineral fertiliser on different agricultural systems both, at long and short-time, have found decreases in pH values (Ling et al., 2017; Verdenelli et al., 2019; Pan et al., 2020; Widdig et al., 2020; Bebber and Richards, 2022). Although increases in the pH values as observed here, has also been found in some experiments (Luo et al., 2017; Craig et al., 2021). These results suggest that responses can vary across ecosystem types because of different soil type, application dose, nitrification process, climate, irrigation, timing, etc. (Tian and Niu, 2015; Wei et al., 2017; Pan et al., 2020).

Regarding the fungal community, the predominant phylum in our study was Ascomycota, in accordance with several authors that showed this phylum as one of the most diverse and ubiquitous phyla of eukaryotes predominating in agroecosystems and whose principal role is decomposing organic substrates (Lienhard et al., 2014; Paungfoo-Lonhienne et al., 2015; Francioli et al., 2016; Wang et al., 2017; Pan et al., 2020). In accordance, we found a positive correlation between this phylum and total carbon in soil. The fungal phylum more negatively affected by the chemical fertilization in our study was Rozellomycota. Yu et al. (2020) also found that this phylum decreased with chemical fertilization (NPK) in a rice-wheat cropping system. By contrast, Muneer et al. (2021) and Ma et al. (2022) found the highest percentage of this phylum under the treatment with higher NPK input in a pomelo orchard and rice yield, respectively. Ali et al. (2022) reported that the significant decrease in the relative abundance of Rozellomycota was mainly attributed to the alteration in soil enzyme activities due to the fertilization, a result which is in line with our findings. The relative abundance of the rest of the most abundant fungal phyla was not affected by fertilization in our study. Regarding the fungal functional guilds, we could observe that only the abundance of mycorrhizal fungi and endophytes guilds, were significantly affected by fertilization. Chemical fertilization decreased the abundance of arbuscular mycorrhizal fungi (AMF). In agreement to our results, Paungfoo-Lonhienne et al. (2015) also found that nitrogen fertilizers negatively impacted on arbuscular mycorrhizae in a sugarcane crop system, suggesting that chemical fertilizers could cause deleterious effects on soil and plant functions. The AMF are one of the main components of the soil microbiota in most agroecosystems because of their important role in improved nutrient uptake and plant growth (Smith and Read, 2008). In general, it is well-stated that chemical fertilisation reduces the AMF species richness and alters their community composition (Wang et al., 2009; Alguacil et al., 2010). Although it is well-know that in presence of high nutrient availability the functionality of the AM symbiosis decreases (Yang et al., 2018), the mechanisms determining the decrease of taxonomic diversity and the change in their community composition are mostly unknown. However, it has been suggested that these changes could be caused by differential requirements for nutrients across AMF species (Treseder and Allen, 2002; Treseder et al., 2018).

We observed that the predictive functional composition of the bacteria associated with the plants growing under chemical fertilization treatments was significantly different from that of the control and organic fertilization, and both treatments clustered together thus showing similar bacterial functionality. In accordance to previous studies (Fierer et al., 2012a; Fierer et al., 2012b), the variation of bacterial functional composition by different N fertilization treatments was closely related to the shifts in the bacterial taxonomic community composition. The bacterial community under chemical fertilization, mainly that resulting from highest doses of N (IT4), showed the lowest relative abundances of functions related to amino acid metabolism, biosynthesis of secondary metabolites, lipids, cofactors and vitamins and carbohydrate metabolism, cell motility and cellular processes and signalling such as sporulation. These bacterial functions could indicate a less sustainable soil microbial community structure and lower microbial functionality in general, in contrast to results obtained when applying organic amendments (Ling et al., 2016). On the other hand, the nitrogen is a key driver of soil microbial community composition (Fierer et al., 2013). The knowledge on microbial N cycling genes in an agroecosystem is crucial to decipher the N cycling in them (Cuartero et al., 2022). In this respect, we found that particular chemical fertilization treatments (IT3 and IT4) significantly increased the abundance of genes contributing to ammonia oxidation (*amoA*, *amoB*, *amoC*), that are part of the nitrification process and important for reducing nitrate leaching and emissions of NO and N_2_O (Liu et al., 2017). Therefore, among the detected genes, *amoA*, *amoB*, *amoC* were the most important genes contributing to the variation in nitrogen cycling related to chemical fertilization, suggesting that ammonia-oxidizers were more sensitive to fertilization than nitrogen-fixers and denitrifiers (Sun et al., 2015). Also, an increase in the abundance of the denitrification-related gen *nirK* was found in the chemical fertilization treatments. The main reason for this may be that the metabolite of nitrification, nitrate, which is the substrate for denitrification, so the abundance of genes involved in denitrification, especially the *nirK* gene, also have high contribution of variation in nitrogen cycling community abundance (Sun et al., 2015). On the other hand, we found that the application of compost tea had no effect on the abundance of predictive pathways involved in nitrogen cycle in purslane rhizosphere. Other studies had found increases in the gene expression associated with metabolism, environmental adaptation and immune system with the application of compost tea in cotton (Luo et al., 2022). Therefore, we suggest that the application doses of this organic treatment probably may have been below a threshold concentration to elicit a distinct microbial functional response compared to the inorganic fertilization treatments where different increments were tested.

In conclusion, this study provides the first insights into the response of soil microbial community to different fertilizer types and doses in the purslane crop, showing that in addition to increasing shoot biomass and producing some changes in soil properties such as pH and soil enzymatic activities related to C, N and P biogeochemical cycles, also produce changes in the bacterial and fungal community composition, in spite of the short period of the experiment (3 months). The majority of chemical fertilization treatments decreased the fungal and bacterial diversity as well as some predictive bacterial functional pathways, synonymous with the loss of microbial functioning. However, in order to get stronger evidence that supports the found pattern, longer time-frame experiments that ideally include sampling across different seasons are needed.

## Data availability statement

The datasets presented in this study can be found in online repositories. The names of the repository/repositories and accession number(s) can be found in the article/**Supplementary Material**.

## Author contributions

AC: Methodology, data curation, formal analysis. JP: Conceptualization, methodology, data curation, investigation, funding acquisition, supervision, validation. AL-G: Software, supervision. MR-V: Software. AD: Methodology. MR: Supervision. SP: Validation, visualization. MA: Data curation, funding acquisition, project administration, writing- reviewing and editing. All authors contributed to the article and approved the submitted version.
