# Supplementary material for: Effects of inorganic and compost tea fertilizers application on the taxonomic and functional microbial diversity of the purslane rhizosphere
Source: Front Plant Sci. 2023 Apr 20;14:1159823. doi: 10.3389/fpls.2023.1159823 (PMC10159062; doi:10.3389/fpls.2023.1159823)
Supplement: Supplementary file 1 [file DataSheet_1.docx]

**Effects of inorganic and compost tea fertilizers application on the taxonomic and functional microbial diversity of the purslane rhizosphere**

Carrascosa A^1^, Pascual JA^1^, López-García A^2^, Romo-Vaquero M^3^, De Santiago A^4^, Ros M^1^, Petropoulos SA^5^, Alguacil MM^1*^

*Corresponding author: Alguacil, MM. E-mail: mmalguacil@cebas.csic.es

**SUPPLEMENTARY DATA**

| Table S1. Shoot Biomass and nutrients content of purslane plants grown under five different fertilization treatments. | | | | |
| --- | --- | --- | --- | --- |
| **Treatments** | **Shoot fresh biomass (g)** | **N (g/Kg)** | **P (g/Kg)** | **K (g/Kg)** |
| Control | 10.1 ± 1.9 a | 10.9 ± 0.8 a | 13.2 ± 1.3 c | 41.1 ± 3.0 |
| IT1 | 57.6 ± 7.2 c | 16.0 ± 1.6 ab | 4.0 ± 1.1 ab | 40.5 ± 7.1 |
| IT2 | 61.6 ± 7.9 cd | 18.6 ± 4.2 b | 5.3 ± 2.6 b | 41.3 ± 7.5 |
| IT3 | 64.5 ± 6.4 cd | 19.2 ± 3.4 b | 5.3 ± 1.0 b | 40.1 ± 4.9 |
| IT4 | 82.4 ± 18.0 d | 29.9 ± 6.9 c | 2.7 ± 0.4 a | 36.1 ± 2.1 |
| OT | 33.1 ± 3.6 b | 15.0 ± 1.0 ab | 5.8 ± 0.9 b | 42.3 ± 7.1 |
| **ANOVA**  **F value (P value)** | **31.38 (<0.001)** | **11.76 (<0.001)** | **28.57 (<0.001)** | **0.58 (ns)** |
| Mean±standard deviation, n=4. Significance of effects of treatments on the measured variables is also shown (F-values (P-values)). Values in columns sharing the same letter do not differ significantly (P<0.05) as determined by the Tukey HSD test.     \| Table S2. Values of bacterial ASVs richness (S) and Shannon-Wiener diversity index (H´) from rhizosphere soil of purslane under different treatments. \| \| \| \| --- \| --- \| --- \| \| **Treatment** \| **S** \| **H´** \| \| Control \| 671 ± 34 ab \| 5.82 ± 0.09 bc \| \| IT1 \| 681 ± 77 ab \| 5.85 ± 0.18 bc \| \| IT2 \| 656 ± 50 ab \| 5.52 ± 0.50 ab \| \| IT3 \| 720 ± 31 bc \| 5.93 ± 0.09 c \| \| IT4 \| 607 ± 41 a \| 5.48 ± 0.21 a \| \| OT \| 752 ± 17 c \| 6.01 ± 0.05 c \| \|  \|  \|  \| \| **F values (P values)** \| **4.86 (0.006)** \| **3.71 (0.019)** \| \| N300 (IT1), N300P200 (IT2), N300P200K200 (IT3), N600 (IT4) and compost tea (OT) treatments. Data represent Mean ± standard deviation, n=4, except for treatment IT2 (n=3). Values in the same column followed by the same letter are not significantly different according to Tukey´s HSD-test (*P*<0.05). Significance of effects of treatments on the measured variables is also shown (F-values (P-values)). \| \| \|  \| Table S3. Values of fungal ASVs richness (S) and Shannon-Wiener diversity index (H´) from rhizosphere soil of purslane under different treatments. \| \| \| \| --- \| --- \| --- \| \| **Treatment** \| **S** \| **H´** \| \| Control \| 202 ± 21 c \| 3.88 ± 0.17 c \| \| IT1 \| 161 ± 16 ab \| 3.50 ± 0.23 ab \| \| IT2 \| 153 ± 4.0 a \| 3.43 ± 0.19 a \| \| IT3 \| 204 ± 27 c \| 3.84 ± 0.10 c \| \| IT4 \| 159 ± 11 a \| 3.58 ± 0.24 a \| \| OT \| 187 ± 22 bc \| 3.81 ± 0.29 bc \| \|  \|  \|  \| \| **F values (P values)** \| **5.56 (0.003)** \| **3.02 (0.039)** \| \| N300 (IT1), N300P200 (IT2), N300P200K200 (IT3), N600 (IT4) and compost tea (OT) treatments. Data represent Mean ± standard deviation, n=4, except for treatment IT2 (n=3). Values in the same column followed by the same letter are not significantly different according to Tukey´s HSD-test (*P*<0.05). Significance of effects of treatments on the measured variables is also shown (F-values (P-values)). \| \| \| \|  \|  \|  \| | | | | |

Table S4. Relative abundance of pathways predicted by PICRUSt that were significantly different in the bacterial communities of purslane rhizosphere under five different fertilization treatments according to Kruskal-Wallis test. Mean±standard deviation, n=4. Significance of effects of treatments on the pathways is also shown (F-values (P-values)). Values in columns sharing the same letter do not differ significantly (P<0.05) as determined by the Tukey HSD test.

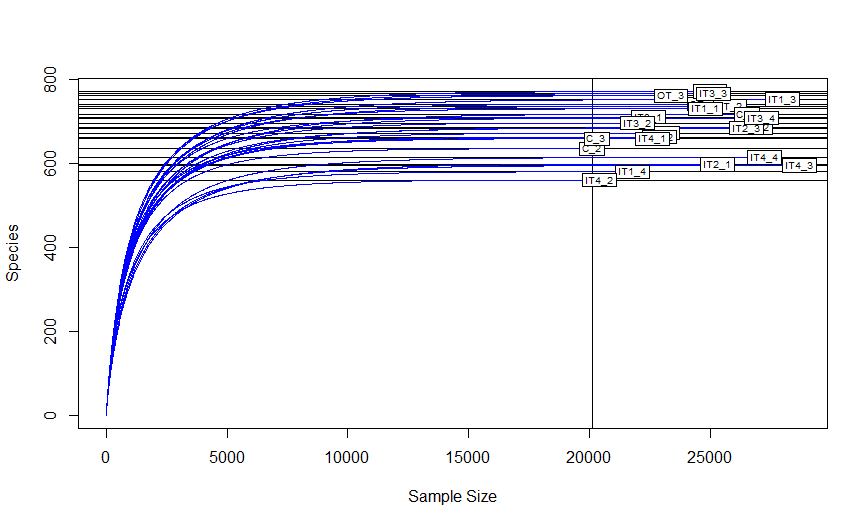


**Figure S1A.** Rarefaction curves on the bacterial ASV dataset at sequencing average depth of about 24,395 sequences per sample


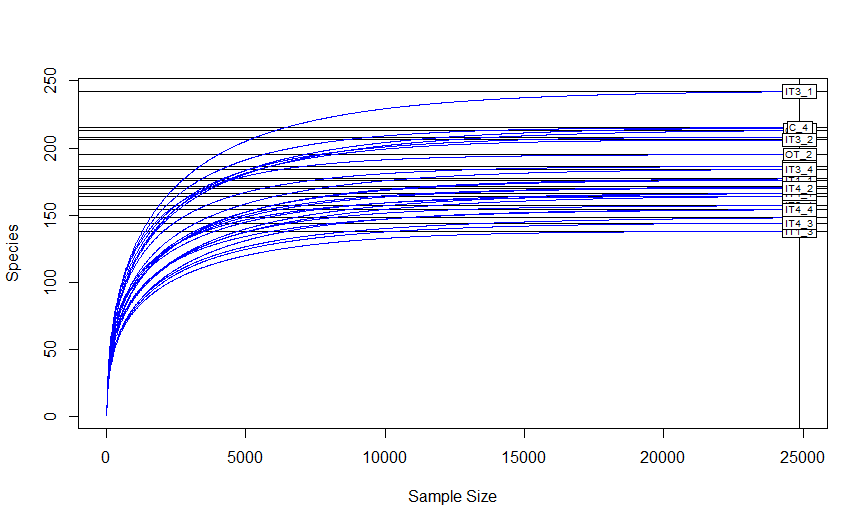


**Figure S1B.** Rarefaction curves on the rarefied fungal ASV dataset at 24,889 sequences per sample

**Fig S2A.** Relative abundances of the dominant bacterial phyla of the rhizospheric soil of purslane under different fertilization treatments. For each phylum, significant differences were assessed by the Tukey´s post-hoc test calculated at *p <* 0.05 and indicated by an asterisk, *p < 0.05; **p < 0.01; ***p < 0.001.

**Fig S2B.** Relative abundances of the dominant fungal phyla of the rhizospheric soil of purslane under different fertilization treatments For each phylum, significant differences were assessed by the Tukey´s post-hoc test calculated at *p <* 0.05 and indicated by an asterisk, *p < 0.05; **p < 0.01; ***p < 0.001.

**Fig. S3A.** Relative abundances of functional groups (saprotrophs, plant pathogens, parasites, endophytes and mycorrhizal fungi) predicted using guild database Fungal Traits for the fungal communities of the rhizospheric soil of purslane plants under different fertilization treatments.

**Fig. S3B.** Relative abundances of functional groups (saprotrophs, plant pathogens, parasites, endophytes and mycorrhizal fungi) predicted using guild database Fungal Traits for the fungal communities of the rhizospheric soil of purslane plants under different fertilization treatments. For each treatment, bars followed by the same letter are not significantly different according to Tukey's HSD-test (p < 0.05). Bars represent standard error.


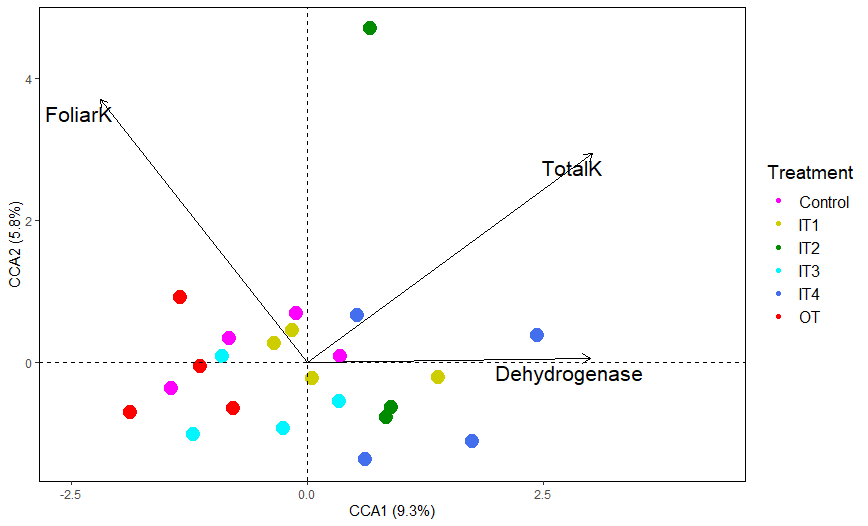


**Fig. S4A**. Canonical correspondence analysis (CCA) on rhizosphere bacterial communities of purslane plants under different fertilization treatments, at ASV level. The explanatory variables (arrows) are those which better explain the community’s variance in the CCA model after a forward selection procedure.


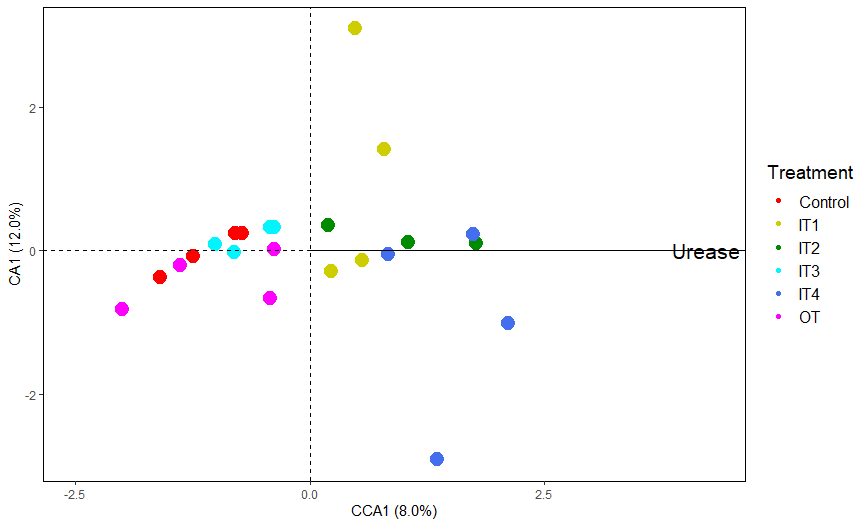


**Fig. S4B.** Canonical correspondence analysis (CCA) on rhizosphere fungal communities of purslane plants under different fertilization treatments, at ASV level. The explanatory variable (arrows) is those which better explain the community’s variance in the CCA model after a forward selection procedure.
